# Sudden Cardiac Arrest During a Sedated Cardiac Magnetic Resonance Study in a Nonsyndromic Child with Evolving Supravalvar Aortic Stenosis Due to Familial ELN Mutation

**DOI:** 10.1007/s00246-022-03089-3

**Published:** 2023-02-15

**Authors:** Dor Markush, Pedro A. Sanchez-Lara, Katheryn Grand, Robert Wong, Ruchira Garg

**Affiliations:** 1 Guerin Family Congenital Heart Program, Smidt Heart Institute, Cedars-Sinai Medical Center, Los Angeles, CA USA; 2 Department of Pediatrics, Cedars-Sinai Medical Center, Los Angeles, CA USA; 3 Department of Medical Genetics, Cedars-Sinai Medical Center, Los Angeles, CA USA; 4 Department of Anesthesiology, Cedars-Sinai Medical Center, Los Angeles, CA USA

**Keywords:** Supravalvar aortic stenosis, Elastin, Elastin arteriopathy, Williams syndrome, Sudden cardiac arrest

## Abstract

Supravalvar aortic stenosis (SVAS) is a less common but clinically important form of left ventricular outflow tract obstruction, and commonly associated with Williams syndrome (WS). SVAS outside of WS may also occur sporadically or in a familial form, often with identifiable mutations in the elastin (*ELN*) gene. While risk of sudden cardiac death in patients with SVAS has been extensively described in the context of WS, less is known about risk in patients with isolated SVAS. We report a case of a nonsyndromic two-year-old boy with evolving manifestations of SVAS who developed sudden cardiac arrest and death during a sedated cardiac magnetic resonance imaging study. A strong family history of SVAS was present and targeted genetic testing identified an *ELN* gene mutation in the boy’s affected father and other paternal relatives. We review risk factors found in the literature for SCA in SVAS patients and utilize this case to raise awareness of the risk of cardiac events in these individuals even in the absence of WS or severe disease. This case also underscores the importance of genetic testing, including targeted panels specifically looking for *ELN* gene mutations, in all patients with SVAS even in the absence of phenotypic concerns for WS or other genetic syndromes.

## Introduction

Congenital supravalvar aortic stenosis (SVAS) is relatively rare, accounting for less than 0.5% of all congenital heart defects, and is the least common form of congenital left ventricular outflow tract obstruction [1]. It is classically associated with Williams syndrome (WS), also known as Williams-Beuren syndrome [2]. However, isolated SVAS without phenotypic features of WS can also occur sporadically or as a familial inherited isolated mutation in the elastin (*ELN*) gene [3–5]. The increased risk of periprocedural sudden cardiac arrest in patients with WS and SVAS is well described [6–11]. However, less is known about the risk of acute cardiac events in nonsyndromic patients with SVAS, especially in the absence of severe disease [11, 12].

## Case

This was a 2-year-old otherwise healthy and active toddler with no cardiorespiratory symptomatology referred for murmur evaluation. Prior history noted an echocardiogram performed in the postnatal period which showed mild peripheral pulmonary stenosis and no aortic stenosis. At 17 months of age, a newly appreciated murmur prompted referral with no antecedent cardiorespiratory symptoms or medical concerns. Family history revealed multiple family members with SVAS, including in the patient’s father, paternal uncle, and paternal grandfather, all of whom required surgical repair in childhood ranging from ages 3 to 20 years. Echocardiography at this time demonstrated normal aortic dimensions with no stenosis, mild right pulmonary artery stenosis (diameter Z-score − 2.4, peak Doppler gradient 29 mmHg), and normal left pulmonary artery size and flow (Z-score + 1.8, peak gradient 13 mmHg). A 6 month interval follow up echocardiogram (now 2.5 years of age) was technically challenging due to patient movement but demonstrated an improving right pulmonary artery gradient (peak of 20 mmHg). The echocardiogram also suggested evolving mild narrowing at the supravalvar aortic area with some size discrepancy between the sinotubular junction and the ascending aorta (sinotubular junction Z-score − 1.7, ascending aorta Z-score + 1.9). Peak Doppler velocity and gradient across this area was only borderline increased at 1.9 m/s and 15 mmHg, respectively. Biventricular size and systolic function were normal and there was no ventricular hypertrophy. EKG showed sinus rhythm with normal voltages for age and a normal corrected QT interval of 388 ms. The patient was clinically asymptomatic, nondysmorphic, and meeting normal growth and developmental parameters.

Referral was made for genetic evaluation given the significant family history and evolving cardiac manifestations. Previous genetic testing showed normal findings on prenatal karyotype and chromosome microarray. An extensive congenital heart disease panel (42 gene, Invitae) was ordered on the patient’s father in context of the extensive paternal history of SVAS, which demonstrated the presence of an isolated heterozygous pathogenic *ELN* gene mutation (c.2044G>T, p.Gly682*). While genetic testing results were pending, to better delineate the anatomy in this patient with a malignant family history for hemodynamically important SVAS and suboptimal echocardiography windows, cardiac magnetic resonance (CMR) was pursued.

The CMR was performed a few days after results became available. General anesthesia was administered by a pediatric cardiac anesthesiologist. Inhalation induction with peripheral intravenous line placement and endotracheal intubation occurred with no issues, with intubation utilized to permit suspension of respirations during CMR image acquisition. Cisatracurium was used for paralysis and anesthetic effect maintained using sevoflurane. There were no significant hemodynamic disturbances or electrocardiogram changes until the end of the examination, when the patient developed sudden ventricular fibrillation and acute cardiac arrest. CPR was initiated immediately and extensive resuscitative measures were performed including administration of various code medications and multiple attempts at defibrillation. ECMO was discussed but was not possible for small children in our institution at the time. Resuscitative efforts were not successful and the patient expired.

**Fig. 1:**
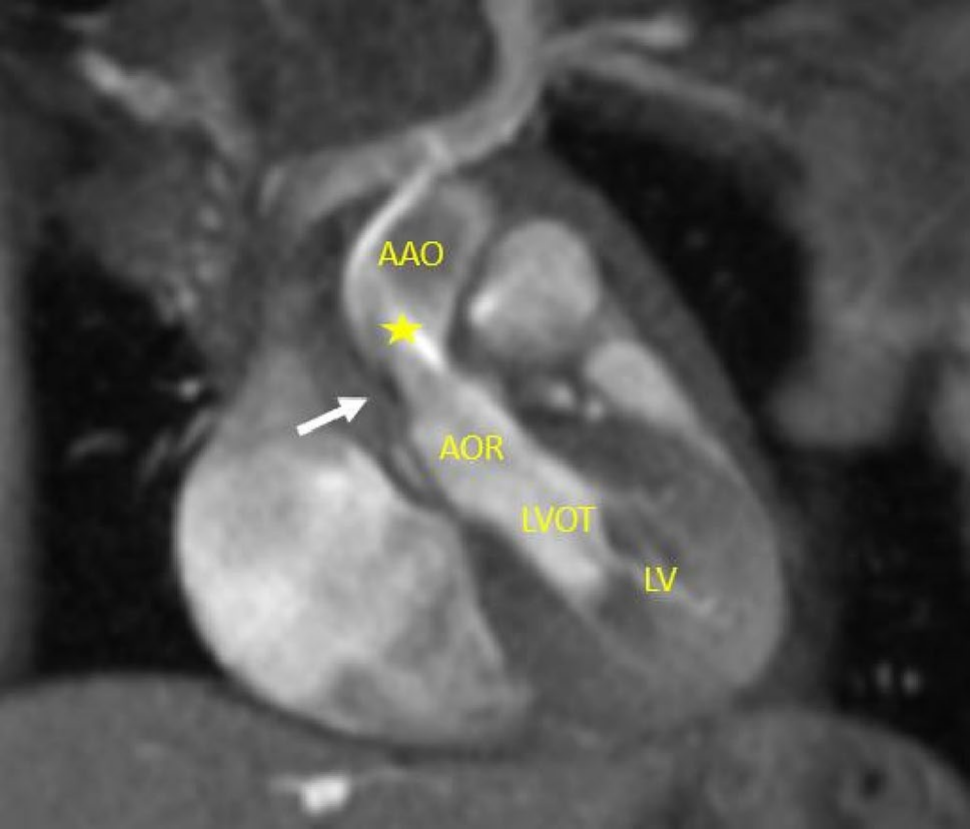
CMR view of the LVOT. Systolic still frame from the patient’s CMR, LVOT CINE view in the coronal plane. Narrowing is seen at the aortic sinotubular junction (white arrow) with a turbulent dephasing jet which starts at this area (below the yellow star), consistent with supravalvar aortic stenosis. *AAO* ascending aorta, *AOR* aortic root, *LV* left ventricle, *LVOT* left ventricular outflow tract

Available CMR images obtained were consistent with mild-to-moderate SVAS (Fig. 1), mild-to-moderate proximal right pulmonary artery hypoplasia, normal left pulmonary artery dimension, symmetric branch pulmonary artery flow distribution, no right ventricular hypertrophy, mild left ventricular hypertrophy, and normal biventricular size and systolic function. The family declined an autopsy.

## Discussion

SVAS is characterized by narrowing of the supravalvar aortic area, and is typically associated with WS, a congenital multisystem disorder involving the cardiovascular, connective tissue, and central nervous systems caused by a large 1.5–1.8 Mb deletion on chromosome 7q11.23 incorporating up to 27 different genes including *ELN* [2, 13–16]. SVAS is the most common cardiac lesion in WS, estimated to occur in approximately 50% of patients, and can commonly coexist with supravalvar or peripheral branch pulmonary stenosis in these patients [17]. Nonsyndromic SVAS may occur outside of WS with identifiable genetic cause in the context of a familial autosomal-dominant inheritance of *ELN* gene mutation or as a sporadic mutation [18, 19]. Histologically, the *ELN* gene mutation results in reduced elastic tissue by formation of broken and disorganized elastin fibers, increased collagen content, and hypertrophied smooth muscle cells, all of which can lead to reduced elasticity and arterial narrowing [20, 21]. Hence, the term Elastin Arteriopathy (EA) has been coined to describe the spectrum of these associated conditions [4]. Not all patients with SVAS have an *ELN* mutation [22], and whether presence of an *ELN* mutation translates into worse disease or a higher risk profile is not fully clear and deserves further study. While there is evidence that some patients with isolated mutations in *ELN* may have less dysfunctional elastin compared to patients with WS who have deletion of the entire *ELN* gene [16, 22, 23], it appears that most *ELN* mutations result in functional haploinsufficiency and thus similar vascular histopathological findings to WS [22]. Nevertheless, significant variable expressivity, reduced penetrance, and varying degrees of clinical severity are seen in patients with EA, with some ranging from asymptomatic carriers never requiring cardiovascular interventions to others displaying early severe disease leading to death in early infancy [22, 24]. Even among cohorts with the same *ELN* mutation, significant intra- and inter-familial variation in phenotype and disease severity can exist [22].

Patients with SVAS and WS are at increased risk for sudden cardiac death compared to the general population [5]. The preponderance of literature on cardiac arrest in SVAS patients is in those with WS, with particular emphasis on the periprocedural period with invasive procedures such as catheterizations, operations, or other noncardiac surgeries [5–11, 25–29]. The hemodynamic burdens from supravalvar aortic and/or pulmonary stenosis, higher incidence of coronary artery abnormalities, and repolarization abnormalities such as QTc prolongation, are some of the contributing factors. The risk of SCD in non-WS patients with SVAS, however, is less well defined and has likely historically been underestimated as data in non-WS SVAS patients are more sparse [11, 12]. We conjecture that past reports with similar EA phenotypes may have batched WS and non-WS patients into same cohorts. It is also possible that non-WS patients with isolated SVAS are less likely to require invasive procedures, or have a lower risk profile for sudden cardiac arrest, although this is unknown and warrants further investigation.. We are also unaware of any prior report of SCD in a non-WS patient during a non-invasive sedated diagnostic study, which is interesting given the common need for diagnostic evaluation often necessary in these patients [30]. While most procedures can be undertaken even in patients with WS without significant adverse events [9, 28, 29], the anesthesia and periprocedural period appears to confer a higher risk for cardiac compromise. Changes in hemodynamic conditions coupled with the arterial obstructive lesions in these patients can result in myocardial ischemia and subsequent sudden cardiac arrest. Sudden cardiac arrest in WS has even been reported during brief noninvasive procedures such as contrast enhanced CT [31].

Available data on risk for SCD in childhood SVAS has also primarily focused on WS patients. High anesthetic risk factors include age < 3 years, history of cardiovascular event or arrhythmia, more than moderate bilateral outflow tract obstruction severity, SVAS gradient > 40 mmHg with presence of left ventricular hypertrophy, known coronary artery involvement, and prolonged QTc interval [25, 26]. Data in children with isolated *ELN* mutations who do not have WS is limited. A recent single institution retrospective review examining risk factors for periprocedural complications in children with various types of EA did include a small cohort of nonsyndromic patients with SVAS (12% of all patients) [11]. In this review, cardiac arrest occurred in 5% of total procedures, all of whom were < 3 years old and had significant biventricular outflow tract obstruction (BVOTO), particularly when gradients across both sides of the heart approached > 40–50 mmHg [11]. Hemodynamic alterations during anesthesia are typically well tolerated in normal children but may be enough to precipitate cardiovascular collapse in children with EA and BVOTO. The presence of BVOTO and presumed resultant hypertrophy likely results in a tenuous myocardial oxygen supply versus demand relationship, which may be further strained in the setting of coronary artery anomalies [11, 32]. Unfortunately detecting the presence or characterization of coronary abnormalities is often not possible by echocardiography, especially in small children, and therefore commonly only noted by direct visual inspection in the operating room or on postmortem autopsy [33]. A high index of suspicion for coronary abnormalities should thus be maintained in all EA patients. However, sudden cardiac arrest can occur even in the absence of coronary issues, although this is usually in the context of severe outflow obstruction [11, 28, 31].

Importantly, while increased risk appears to be associated with coronary abnormalities or significant outflow tract obstruction (especially bilateral) [5, 11, 34], it can be clinically challenging to predict. With the exception of age (just under 3 years), our patient manifested no additional high risk features described for the WS SVAS population. However, the tragic outcome demonstrates the difficulty in predicting risk of cardiac decompensation in such patients, and that risk profile may also not be dependent on disease severity. While all attempts should be made to avoid non-essential sedated procedures in these patients, even if noninvasive in nature, many will nonetheless require diagnostic and interventional workup and treatment. It is strongly recommended that sedated procedures in children with EA, especially infants and toddlers, be performed at larger tertiary centers with highly trained professionals and expertise in caring for such patients, including ability for rapid ECMO cannulation. In places where cardiac CTA may be performed without or with minimal sedation even in small children, this can also be considered prior to sedated options. The risk–benefit ratio for any diagnostic test or anticipated sedation should be thoroughly considered, and frank discussions regarding the risks and benefits should occur with the family. Additionally, given the imaging-based disease progression noted even over a short 3 month period between the last echocardiogram and CMR, we suggest that every patient undergo a repeat EKG and echocardiogram for up to date assessment ideally within a month prior to a sedated procedure.

Genetic testing for patients with SVAS is an important consideration and likely has traditionally been underevaluated in nonsyndromic patients. First-line genetic testing methods typically include karyotype, FISH, or chromosomal microarray (CMA) [35]. CMA, in particular, will identify many of the genetic abnormalities associated with CHD, including WS. However, it generally lacks the sensitivity to detect isolated single gene mutations. Targeted gene panels are require, but are more widely available today and therefore promise to improve identification of *ELN* mutations in patients with SVAS. Newer, even broader molecular approaches such as Whole Exome Sequencing may also be useful when clinical features are subtle or non-specific [36]. Increased awareness across the scientific community for *ELN* mutations in patients with SVAS, even in the absence of concern for WS, is necessary to improve testing and identification of nonsyndromic patients. We recommend that all patients with SVAS (and/or peripheral pulmonary stenosis which does not resolve in the first couple years of life) undergo thorough genetic evaluation, including targeted testing for isolated *ELN* mutations. Improved identification of patients with EA would help optimize counseling, inform cascade family genetic testing, and prompt coordination of periprocedural multidisciplinary care, all of which can hopefully reduce adverse outcomes in this fragile and important patient population.
